# Diagnosis and Management of a Young Girl With Tumor Necrosis Factor Receptor Associated Periodic Syndrome (TRAPS) Linked to a Novel Mutation

**DOI:** 10.7759/cureus.10766

**Published:** 2020-10-02

**Authors:** Eleni Klinaki, Adrianos Nezos, Athanasios G Tzioufas, Maria N Tsolia, Despoina N Maritsi

**Affiliations:** 1 Rheumatology, "P. & A. Kyriakou" Children's Hospital, Medical School, National and Kapodistrian University of Athens, Athens, GRC; 2 Pathophysiology, Medical School, National and Kapodistrian University of Athens, Athens, GRC; 3 Infectious Diseases, “P. & A. Kyriakou” Children’s Hospital, Medical School, National and Kapodistrian University of Athens, Athens, GRC; 4 Rheumatology, “P. & A. Kyriakou” Children’s Hospital, Medical School, National and Kapodistrian University of Athens, Athens, GRC

**Keywords:** autoinflammatory diseases (aids), tumor necrosis factor receptor-associated periodic syndrome (traps), tnfrsf1a mutations, clinical phenotype, interleukin-1β inhibitor

## Abstract

A nine-year-old girl with a two-month history of fever and generalized malaise, along with intermittent abdominal pain, immigrant myalgia, throat pain, anorexia, and long-standing failure to thrive, was admitted to our department for further investigation and treatment. Detailed medical history revealed recurrent inflammation attacks from a very young age and a heavily burdened family history. Tumor necrosis factor receptor-associated periodic syndrome (TRAPS) was highly suspected. Genetic screening was performed and several members of the family were found to be carriers of C73Y mutation in exon 3, which is a novel tumor necrosis factor superfamily receptor 1A (TNFRSF1A) mutation. The girl was treated with an interleukin-1β inhibitor, canakinumab, which induced immediate and complete remission of disease that interestingly lasted for a long period even after medication discontinuation.

## Introduction

Autoinflammatory diseases (AIDs) are a genetically heterogeneous group of disorders of the innate immune system caused by mutations of genes encoding proteins, which play a pivotal role in the regulation of the inflammatory response. The most usual and well-characterized group of autoinflammatory syndromes are those associated with ‘‘periodic fevers’’, where episodes of seemingly unprovoked sterile inflammation without high-titer autoantibodies or antigen-specific T lymphocytes, manifest mainly as recurrent fever and disease-specific patterns of organ inflammation. The main inherited periodic fever syndromes are monogenic and are caused by highly penetrant genetic variants in single genes [1,2]. Among them, the tumor necrosis factor receptor-associated periodic syndrome (TRAPS) is the most common autosomal dominant disorder in Europe, albeit it is a very rare disease with an estimated prevalence of about one per million [3,4]. It was first described as “familial Hibernian fever” in 1982 [5] but was renamed to TRAPS when the genetic basis of this condition was discovered. The disease is caused by mutations in the tumor necrosis factor (TNF) superfamily receptor 1A (TNFRSF1A) gene, which encodes the p551A receptor of TNF (TNFR1) [6]. The pathogenesis of TRAPS seems to involve not only the TNF receptor but also the dysregulation in the secretion of interleukin (IL)-1 and IL-6, along with oxidative stress [7]. TRAPS is a pleiomorphic condition characterized by attacks of fever in most cases, accompanied by a variety of non-specific symptoms such as abdominal pain, pleurisy, lymphadenitis, skin rash, eye manifestations, myalgia/arthritis, and fatigue. During the flares, laboratory testing reveals neutrophilic leukocytosis and a marked acute phase response. The most severe complication is AA-type serum amyloidosis, observed in ~14% of the patients [8]. The remarkable clinical diversity of TRAPS is linked to different TNFRSF1A mutations, therefore, diagnosis relies on high clinical suspicion supported by genetic testing [3].

Herein we present the case of a nine-year-old girl with singular symptoms compatible with TRAPS diagnosis, which was genetically enhanced and subsequently confirmed, along with treatment and disease course.

## Case presentation

A petite nine-year-old girl (somatometric features <3rd centile), presenting with a two-month history of fever and generalized malaise, associated with intermittent abdominal pain, immigrant myalgia, and throat pain at disease onset, was admitted to our department for further investigation and treatment. A physical examination along with laboratory and imaging testing revealed elevated inflammatory markers [white blood cell count 23.200/μl (neutrophils: 81%), erythrocyte sedimentation rate (ESR) 117 mm/h, c-reactive protein 261 mg/L, ferritin 421 ng/ml, fibrinogen 6.21 g/L, serum amyloid A 590 mg/L], anemia (hemoglobin 9.9 g/dl), mesenteric lymphadenitis, myositis/fasciitis of the left major gluteal muscle and corneal sediments due to uveitis. No clear source of infection was detected. On close questioning, we realized that the patient had two more admissions in the past for prolonged pyrexia. At the age of 12 and 26 months, she was hospitalized for persistent fever of unknown origin. At that time, laboratory and imaging testing were inconclusive as the only abnormal finding was the elevated inflammatory markers (leucocytosis and raised ESR). Additionally, at the age of seven and eight years, the child presented with arthritis, which resolved automatically. Moreover, short-term fever episodes of unknown origin without any accompanying symptoms, with a frequency of 1-2 episodes per year, were reported.

Further investigation into our patient’s family history revealed that her father and several members of the parental family manifested similar symptoms without having a clear diagnosis. Her father suffered from arthritis of small and large joints and was treated as seronegative arthritis without remission of symptoms. Moreover, during childhood, he presented with recurrent episodes of fever of unknown origin, as well as abdominal pain; he had been operated twice for adhesive obstructive ileus. Similar symptomatology was reported in other four members of the paternal family. The paternal father had been diagnosed and unsuccessfully treated as ankylosing spondylitis and had been operated for coronary heart disease at a young age. Three out of five paternal brothers and sisters were bearing symptoms of arthritis, serositis, and abdominal pain presenting at a young age.

**Figure 1:**
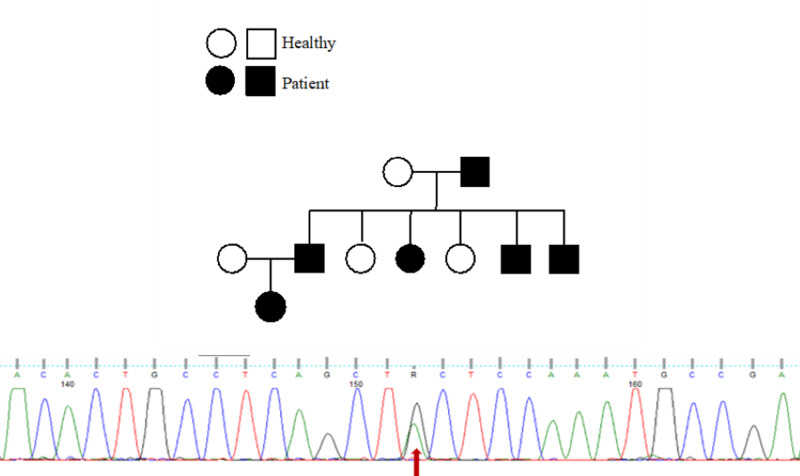
Family pedigree and the Sanger sequencing electropherogram for the exon 3 C73Y mutation (c.305G>A, p.C102Y)

Given the aforementioned findings of the child and the burdened family history of a chronic recurrent inflammatory disease, a hereditary AID was suspected and TRAPS was considered as the most likely diagnosis. Therefore, the child was screened for TNFRSF1A (accession number: NM_001065) gene mutations. Indeed, a novel heterozygous mutation in the cysteine residue located in exon 3, C73Y, as a common name (c.305G>A, p.C102Y). Following positive testing of the child, relatives were recruited, informed consent was obtained and blood samples were collected for targeted genetic examination. The same mutation was identified in five members, two of whom also carried a concomitant R92Q mutation while three members were carriers of the R92Q (Figure 1).

The girl was initially treated with steroids with partial response, followed by administration of interleukin-1β inhibitor, canakinumab, at a dose of 120mg per month (4mg/kg) [2,9,10]. Complete resolution of symptoms and normalization of inflammatory markers was observed after the first dose of the IL-1β inhibitor. No adverse events were reported. Due to unforeseen circumstances, treatment was stopped at 12 months. Interestingly, one year after treatment termination, the child remains completely asymptomatic and presents a normal growth rate.

## Discussion

Over the past 20 years the list of AIDs has been continuously enriched and thorough research has shed light over the pathophysiological pathways and management of these conditions. However, even nowadays, these disorders are very challenging since their diagnosis is mostly based on high index of clinical suspicion. Pediatricians may easily get misled due to the rarity of these diseases, the overlapping signs and symptoms, both between disorders of the inflammatory spectrum and different conditions, as well as the non-pathognomonic findings. As supported by our case report, careful clinical observation of characteristic pattern of inflammation attacks along with disease course and a detailed familial history can provide invaluable information, thereby reducing further delays in diagnosis, not-targeted diagnostic investigations and therapeutic failures.

TRAPS is the second most common hereditary periodic fever syndrome following the familial Mediterranean fever (FMF). It is an autosomal dominant disease associated with the p551A receptor of TNF (TNFR1), encoded by the TNF superfamily receptor 1A (TNFRSF1A) gene on chromosome 12, whose mutations cause the disease [1,6]. The involved gene is composed of 10 exons with the disease-causing mutations, all missense and heterozygous, concentrated into exons 2, 3, 4, and 6, that can be distinguished to high- or low-penetrance ones [1]. The remarkable diversity of clinical phenotypes in TRAPS is linked to the variety of mutations. The high-penetrance mutations, like the rare C73Y mutation found in our case, are located in cysteine-rich N-terminal domains, which are important for the assembly of the receptor’s three-dimensional structure, understood to cause an early disease onset and more severe clinical manifestations [11]. On the contrary, mutations like the R92Q variation may exhibit high inheritance rate but low penetrance impact in the disease phenotype. [8,12]. Although the biological alteration involves the TNF receptor, the pathogenesis of TRAPS also seems to be associated with a dysregulation in the secretion of IL-1 and IL-6, as well as oxidative damage correlated with the mitochondrial production of free radicals [7]. The molecular link between TRAPS and IL-1 has not been fully elucidated. The pathogenesis may vary with each mutation, but it is supported that IL-1 might act as a proinflammatory mediator downstream of TNF, or that aggregates of the misfolded TNF receptors stimulate intracellular signals resulting in enhanced production of IL-1 and other chemokines [13]. In addition, IL-1 is linked to mediating pathology in metabolically driven low-grade chronic inflammation, believed to contribute to disease severity and outcome of conditions like type 2 diabetes, and atherosclerosis [14].

The wide range of clinical manifestations in patients with TRAPS makes diagnosis even more challenging for pediatricians. According to the most recent data reported from the Eurofever/EUROTRAPS registry, the median age of onset was 3.4 years (range 0.8-10.6) and the median disease duration at diagnosis was 13.3 years (6.8-23.2). The mean number of reported episodes was six per year, with a mean duration of episodes lasting eight days [15]. From a previous data analysis of the registry, the dominant symptom was fever (>88%) accompanied by myalgia (85%), abdominal pain (74%), urticarial rash (63%), and ocular involvement (45%, as periorbital edema and conjunctivitis). Lymphadenopathy, eye symptoms, and abdominal pain were more frequent in children. Patients with low-penetrance mutations, such as R92Q, had a non-significant trend towards more oropharyngeal symptoms. Of note, 10% of the study participants had developed AA amyloidosis at diagnosis [3].

Our case patient experienced early disease onset. Fever episodes were characterized by low frequency, and each time she presented with various clinical manifestations. However, the overall clinical manifestations of the patient collect the majority of the TRAPS symptoms’ spectrum. Moreover, most of the episodes were self-limited and recessed without treatment. A similar clinical pattern was present in the rest of the affected relatives with differentiation of findings over the course of time and disease. What is worth mentioning is the fact that besides the certification of a high-penetrance mutation, none of the patients with a long and untreated disease duration developed secondary amyloidosis, which is the most dreaded complication, but two of them suffered from adhesions due to serositis. For the time being, over 100 mutations have been reported and causally associated with the presence of the syndrome. To the best of our knowledge, the C73Y mutation is considered very rare based on the available literature [16]. Therefore, data reported in the present case study strengthen the linkage between this rare genetic variance and TRAPS phenotype of early-onset recurrent inflammation attacks mainly characterized by fever, serositis, and myoskeletal symptoms. Finally, another interesting finding was the fact that the administration of canakinumab induced rapid disease control, while clinical benefits were sustained for long-term after treatment termination.

## Conclusions

TRAPS is a rare hereditary periodic fever syndrome, which is a subgroup of autoinflammatory diseases, characterized by a remarkable diversity of clinical phenotypes, making the diagnosis even more challenging. Herein we present the case of a young girl with recurrent inflammation attacks with long-standing failure to thrive and a heavily burdened family history. Targeted genetic testing revealed a very rare TNFRSF1A gene mutation linked to TRAPS clinical manifestations. Treatment with interleukin-1β inhibitor led to an immediate and complete remission of disease, which interestingly lasted for a long period even after medication discontinuation. 

## References

[1] Martorana D, Bonatti F, Mozzoni P, Vaglio A, Percesepe A (2017). Monogenic autoinflammatory diseases with mendelian inheritance: genes, mutations, and genotype/phenotype correlations. Front Immunol.

[2] Ozen S, Demir S (2017). Monogenic periodic fever syndromes: treatment options for the pediatric patient. Pediatr Drugs.

[3] Lachmann HJ, Rapa R, Gerhold K (2014). The phenotype of TNF receptor-associated autoinflammatory syndrome (TRAPS) at presentation: a series of 158 cases from the Eurofever/EUROTRAPS international registry. Ann Rheum Dis.

[4] Lainka E, Neudorf U, Lohse P (2009). Incidence of TNFRSF1A mutations in German children: epidemiological, clinical and genetic characteristics. Rheumatology.

[5] Williamson LM, Hull D, Mehta R, Reeves WG, Robinson BH, Toghill PJ (1982). Familial Hibernian fever. Q J Med.

[6] McDermott MF, Aksentijevich I, Galon J (1999). Germline mutations in the extracellular domains of the 55 kDa TNF receptor, TNFR1, define a family of dominantly inherited autoinflammatory syndromes. Cell.

[7] Caso F, Rigante D, Vitale A (2013). Monogenic autoinflammatory syndromes: state of the art on genetic, clinical, and therapeutic issues. Int J Rheumatol.

[8] Nezos A, Argyropoulou OD, Klinaki E (2020). Molecular and clinical spectrum of four pedigrees of TRAPS in Greece: results from a national referral center. Rheumatology.

[9] ter Haar NM, Oswald M, Jeyaratnam J (2015). Recommendations for the management of autoinflammatory diseases. Ann Rheum Dis.

[10] Gattorno M, Obici L, Cattalini M (2017). Canakinumab treatment for patients with active recurrent or chronic TNF receptor-associated periodic syndrome (TRAPS): an open-label, phase II study. Ann Rheum Dis.

[11] Chan FK, Chun HJ, Zheng L, Siegel RM, Bui KL, Lenardo MJ (2000). A domain in TNF receptors that mediates ligand-independent receptor assembly and signaling. Science.

[12] Pelagatti MA, Meini A, Caorsi R (2011). Long-term clinical profile of children with the low-penetrance R92Q mutation of the TNFRSF1A gene. Arthritis Rheum.

[13] Savic S, Dickie LJ, Wittmann M, McDermott MF (2012). Autoinflammatory syn-dromes and cellular responses to stress: pathophysiology, diagnosis and new treatment perspectives. Best Pract Res Clin Rheumatol.

[14] Jesus AA, Goldbach-Mansky R (2014). IL-1 blockade in autoinflammatory syndromes. Annu Rev Med.

[15] Gattorno M, Hofer M, Federici S (2019). Classification criteria for autoinflammatory recurrent fevers. Ann Rheum Dis.

[16] Lee RU, Saland S, Sullivan S (2013). Tumor necrosis factor receptor-associated periodic syndrome as a cause of a recurrent abdominal pain in identical twins and description of a novel mutation of the TNFRSF1A gene. J Pediatr Gastroenterol Nutr.

